# Evaluation and optimization of municipal-level health impact assessment policies in China based on the PMC index model

**DOI:** 10.3389/fpubh.2025.1693278

**Published:** 2025-12-15

**Authors:** Ying Zhou, Lin Cheng, Yanshan Zhu, Jun Yuan, Yu Ma

**Affiliations:** 1 Central Office, Guangzhou Center for Disease Control and Prevention (Guangzhou Health Supervision Institute), Guangzhou, China; 2 Institute of Public Health, Guangzhou Medical University & Guangzhou Center for Disease Control and Prevention (Guangzhou Health Supervision Institute), Guangzhou, China

**Keywords:** policy consistency index model, health impact assessment, policy, evaluation, optimization

## Abstract

**Objective:**

This study aims to develop an integrated framework combining text mining with the Policy Modelling Consistency (PMC) index model to evaluate China’s Health Impact Assessment (HIA) policies, thereby advancing the methodological and theoretical framework for policy evaluation in health governance.

**Methods:**

This study focuses on health impact assessment policies issued at the municipal level in China since 2019. Using the NVivo 11 plus text mining tool, high-frequency terms from the policies were extracted to construct a Policy Modeling Consistency (PMC) index model for health impact assessment policies. Quantitative analysis was conducted on 30 policies using nine primary variables and 39 sary variables.

**Results:**

The average PMC index score was 6.89. Among the sample policies, 11 were deemed complete and reasonable, and 19 were considered acceptable. The quality of the policies was at an acceptable level or above. The policies scored highly in terms of policy nature, content, and evaluation, but scored lower in policy timeliness, issuing authority, safeguards, and policy tools. The average concavity index was 3.11, indicating a moderate concavity level. The concavity index indicates that existing policies have established a solid foundation, ensuring the orderly advancement of the pilot program. However, the policies’ intensity and the balance within their internal structure remain inadequate, suggesting that future efforts should focus on strengthening incentive mechanisms and addressing shortcomings.

**Conclusion:**

This study adapts the PMC model and validates its applicability in policy analysis for health impact assessments, providing a replicable framework for evaluating health policies in developing countries. China’s municipal-level health impact assessment policies have effectively safeguarded public health security and advanced the implementation of the “Health in All Policies” approach. However, numerous challenges persist, including an inadequate legal framework, weak oversight mechanisms, imbalanced policy tools, and insufficient interregional collaboration. Based on this, it is recommended that future efforts strengthen top-level design, address the aforementioned key aspects, and systematically advance the optimization and improvement of health impact assessment policies to better align with the strategic needs of China’s health development in the new era, enhance policy effectiveness, and promote the sustained improvement of public health levels.

## 1 Introduction

### 1.1 Global context and problem definition

Health impact assessment (HIA) is a combination of procedures, methods, and tools that can assess the impact of policies, plans, or projects on population health and its distribution (1). The essence of the HIA system is to institutionalize the consideration of health throughout the entire process of public policy formulation and implementation across all sectors. It is a core strategy for implementing the principle of “health in all policies” internationally, reflecting the concepts and requirements of modernizing national governance systems and capabilities (2, 3). Establishing a comprehensive HIA system is a complex and challenging systemic endeavor, with high requirements for both institutional design and technical methodologies. The HIA process spans multiple disciplines and fields, requiring not only the provision of professional, authoritative, and timely policy optimization recommendations but also the establishment of highly coordinated interdepartmental collaboration and management mechanisms.

### 1.2 Literature review and theoretical framework

International scholars have conducted extensive and in-depth research on the formation, development, practical application, and related institutional construction of health impact assessment systems, accumulating rich research findings and practical experience (4, 5). Since China officially launched the pilot program for the Health Impact Assessment (HIA) system in 2021 (6), many cities have actively responded and boldly explored, achieving a series of phased results. However, during the actual implementation process, some urgent challenges and issues have emerged, such as the late start of institutional development, insufficiently sound operational mechanisms, inadequate professional technical capabilities, and limited social influence. These issues have to some extent constrained the full realization of the system’s effectiveness. From the current state of research, studies on China’s HIA system primarily focus on two aspects: first, the experience summary of international policy frameworks, analyzing successful practices from different countries in HIA system development to provide references for system improvement; second, case studies in specific fields, analyzing the implementation effects and existing issues of HIA in specific scenarios (2, 3, 7–9)^.^ However, existing research faces two major limitations. First, there is a lack of systematic compilation and in-depth evaluation of health impact assessment policies at the municipal level in China, hindering a comprehensive understanding of the current implementation status and obstacles to improvement. Second, at the methodological level, studies often focus on macro-level generalizations, with insufficient attention paid to textual quantitative analysis and discourse analysis.

### 1.3 The research value of the Chinese case

Health impact assessment aims to eliminate health risks and safeguard health “red lines,” serving as a core strategy for international implementation of the “Health in All Policies” approach. Some international scholars’ practical experiences and theoretical explorations provide positive and beneficial references and insights for establishing China’s health impact assessment system. Nina Lamprecht et al. conducted a systematic review and revealed a significant increase in health impact assessment (HIA) publications over the past 15 years. However, a substantial gap persists between low/middle-income countries and high-income countries in research output, with the ratio shifting from 6:94 in 2008 to 11:89 today (4). This growth primarily stems from a sharp increase in “research-driven HIAs,” while traditional “stepwise HIA” practices show no clear upward trend. The study also highlights a significant trend: the term “HIA” is being applied more broadly, increasingly used to refer to health impact modeling studies and quantitative health risk assessments. Jabot et al. addressed the critical policy question of how to scientifically and systematically evaluate the effectiveness of Health Impact Assessments (HIAs). Drawing on a French case study and assessment literature, researchers developed a logic model for monitoring and evaluating HIA effectiveness (10). This framework encompasses three dimensions—context, implementation, and governance—highlighting multiple pathways through which HIAs drive change and identifying key success factors. It provides a significant methodological reference for policy evaluation practices concerning HIAs. Haigh, F et al. identified four priority areas for HIA research over the next decade through a mixed-methods approach (international online survey, participatory workshops, and literature review): Institutionalization, Effectiveness, Equity and Participation, and Methodology (11). This provides a clear roadmap and research framework for national HIA development. The study further emphasizes the need for interdisciplinary research approaches, enhanced international comparative case studies, and consideration of diverse knowledge and research methods within HIA. These efforts will drive sustained advancement in the HIA field to address global health challenges and build a healthier, more equitable world. However, the applicability of the above international experiences in China requires further exploration.

Although China’s research in this area started relatively late and remains in its initial and learning phase, it has demonstrated strong vitality on the path toward governance modernization. Given the ongoing advancement of HIA pilot programs, China, as a major representative of developing countries, should prioritize quantitative research on HIA policies. This research can provide a robust theoretical and practical foundation for summarizing and reflecting on China’s pilot programs and for expanding pilot initiatives in other developing countries.

### 1.4 Research gaps and methodology

Policy text quantitative analysis is a technical approach that employs advanced statistical methods to quantify and analyze the content of policy texts. Through data mining and model construction, it can reveal policy characteristics, evolutionary trends, and potential issues, providing a more scientific and objective basis for policy research. The PMC index model has been widely applied in policy text quantitative analysis. It was established by RuizEstrada (12) based on the OmniaMobilis hypothesis, which suggests that when designing policy research models, efforts should be made to broaden the scope and ensure the comprehensiveness of variable indicators (13). Selecting the PMC model over other mainstream policy text analysis methods offers the following advantages: (1) Traditional content analysis scoring frameworks typically focus on frequency counts and single-dimensional scoring of specific keywords or high-frequency terms within policy texts. The PMC model constructs a comprehensive indicator system encompassing multiple primary and secondary variables, enabling deeper assessment of internal consistency and external multidimensional attributes (such as policy nature, effectiveness, domain, tools, etc.), resulting in more comprehensive and systematic analysis. (2) Policy Evaluation Matrices often rely on expert subjective judgments or yield qualitative, fragmented conclusions. The PMC model transforms multidimensional variable information into quantifiable PMC indices through standardized parameter calculations and generates PMC surface plots, making complex policy evaluations more intuitive and objective. (3) The PMC model can rank and classify multiple policies, clearly identifying policy shortcomings or “policy gaps.” This approach aligns well with the objectives of this study, as it not only assesses overall policy effectiveness but also pinpoints key optimization pathways, providing clear and concrete grounds for policy refinement. Given the scarcity of comprehensive quantitative analyses of policy texts related to health impact assessment policies in China, this study employs the Policy Modeling Consistency (PMC) index model to focus on a key question: How do municipal governments translate national health strategies into specific policy actions within the “central-local” governance framework? Using Chinese municipal-level health impact assessment policies as a sample, this study employs NVivo 11 plus text mining technology to extract high-frequency policy terms, constructs a policy quantification evaluation system encompassing 9 primary indicators and 39 secondary indicators, determines index calculation methods and rating standards, and selects representative policies for macro–micro and horizontal-vertical comparative evaluations. Finally, we used PMC surface plots to conduct intuitive evaluation analyses of representative policies to determine whether the currently formulated policies meet the systemic standards of the policies.

## 2 Literature review

In 1999, the World Health Organization (WHO) first proposed the concept of Health Impact Assessment (HIA) in the Gothenburg Consensus: Health Impact Assessment is a series of interrelated procedures, methods, and tools used to evaluate the potential health impacts of a policy, plan, or project on a specific population and their distribution within that population (1). This consensus provided legal legitimacy for HIA as a new field and established it as a formally recognized method (14). Based on the timing of the assessment, HIAs can be categorized into three main types: prospective, real-time, and retrospective (15). Countries differ in terms of regulations, organizational structures, application areas, technical procedures, and implementation participants related to Health Impact Assessment. However, Health Impact Assessment research abroad has become increasingly systematized and comprehensive in its focus, with researchers conducting extensive reviews of its formation, development, practice, and related systems. These reviews cover both macro-level issues such as health promotion consensus or health social determinants and equity, as well as meso-level issues such as interdepartmental health collaboration. For example, Nina Lamprecht et al. conducted a systematic review of relevant literature published between June 2007 and January 2023 on PubMed and the Web to explore the gaps in health impact assessment research between developed and developing countries (4). Natalie Mueller et al. conducted expert consultations and literature reviews to organize, compare, and select reference models related to health impact assessment (5). Luo et al. analyzed 2,215 articles related to health impact assessment in the field of urban planning published between 2012 and 2021 using bibliometric analysis based on the ISI Web database (16). Nkyekyer et al. analyzed five representative health impact assessments in the energy and natural resources sectors in the United States between 2007 and 2016 and evaluated their implementation effectiveness using the Wismar framework (17). Kalel reviewed the literature on the implementation of health impact assessments in the Republic of Kazakhstan and studied the country’s health impact assessment governance system (18).

In 2016, the “Healthy China 2030” Planning Outline, as the action plan for advancing the Healthy China initiative, explicitly stated that “a comprehensive health impact assessment system should be established to systematically assess the impact of various socio-economic development plans and policies, as well as major engineering projects, on health” (19). In 2019, the Basic Medical and Health Care and Health Promotion Law of the People’s Republic of China stipulated that “all levels of people’s governments shall place people’s health in a priority position in strategic development and incorporate health concepts into all policies” and “establish a health impact assessment system and include improvements in citizens’ key health indicators in government performance evaluations” (20), marking the first time that health impact assessment was legally enshrined. In 2021, the National Patriotic Health Campaign Committee Office and the Healthy China Action Promotion Committee Office launched a pilot program nationwide to build a health impact assessment system, systematically advancing the practice and theoretical research of health impact assessment. Some provinces and cities have already issued pilot implementation plans for health impact assessment and outlined specific work objectives and requirements for such assessments. Pilot provinces and cities have also successively issued and implemented relevant work plans (6). Compared with other countries, China’s health impact assessment started relatively late, but the concept gained attention at the national decision-making level upon its introduction and has formed a consensus at the national level, becoming a concrete manifestation of the “big health” philosophy in health and wellness work in the new era. From a research methodology perspective, health impact assessment research primarily focuses on case studies and is mainly concentrated on socio-economic development plans and major engineering projects.

From the perspective of research, health impact assessment research exhibits diverse characteristics. Among them, domestic scholars have produced relatively few research outcomes on health impact assessment policies, with research primarily concentrated in three areas: first, the analysis and insights from policies of other countries. Such research analyzes the relevant policies enacted by other countries to provide insights for China’s policy formulation. For example, the research by Zhao Rui and Yuan Jiaqi et al. reviewed the HIA systems of countries and regions such as the United Kingdom, the United States, Canada, Australia, Sweden, New Zealand, and Thailand, and based on this, provided suggestions and outlooks for the institutionalization of China’s HIA system (7, 8, 21, 22). Second, the current status, experience sharing, and problem suggestions regarding the construction of pilot systems at the national, provincial, and municipal levels. Wang Xufeng summarized the current status and issues of health impact assessment in China at the national, provincial, and municipal levels, and proposed that China should clarify the organizational structure, determine the implementing entities, define the assessment targets and scope, strengthen technical support, and accelerate legislation on health impact assessment (2). Zhang Dongxian and others reviewed the current status of health impact assessment development in China from three aspects: theory, policy and regulations, and practical promotion. They analyzed the main issues affecting its development and proposed targeted recommendations for institutional development from the perspectives of laws and regulations, organizational leadership, coordination mechanisms, capacity building, talent cultivation, the new round of health city creation, and supervision and management (3). Wu Jing and others identified the current issues in China’s health impact assessment system and proposed policy recommendations, including establishing and improving the legal, regulatory, and standard framework for environmental and health issues, refining the technical methods for health impact assessment, and establishing and improving public participation and evaluation systems (23). Zhu Lili et al. conducted interviews with experts and scholars (from government departments such as the National Health Commission and the Ministry of Ecology and Environment, as well as research institutions) who have long been engaged in traffic impact assessment, environmental impact assessment, and other fields to collect data. They analyzed the feasibility of implementing a health impact assessment system in China using grounded theory methods (24). Some studies summarized the pilot experiences of current health impact assessment pilot cities, drawing on assessment practice cases to summarize their institutional development from aspects such as organizational structure, assessment scope, implementation pathways, and technical safeguards (25–29). Third, studies focused on the construction and case applications of health impact assessment mechanisms in specific fields. These studies primarily concentrated on national land use planning and urban planning. For example, Zhang Tianyao et al. explored the cognitive foundation, key issues, and proposed schemes for integrating health impact assessment into territorial spatial planning, and put forward corresponding institutional safeguards and policy recommendations (30). Ding Guosheng et al. conducted a summary review of international health impact assessment systems and their development in spatial planning, and proposed suggestions for reference for health impact assessment in China’s territorial spatial planning (9). Li Xiao, Ding Guosheng, Ge Yifu, and others explored assessment mechanisms and methods by drawing on foreign health city research and assessment methods and combining them with the basic situation of urban planning in China (31–33).

Overall, the following issues exist: (1) Research remains largely confined to introducing international experiences without empirical verification of their applicability to the domestic context. Some studies propose institutional pathways for China by reviewing systems in countries such as the UK, the US, and Canada. (2) Most research relies on macro-level summaries of pilot system development, lacking quantitative analysis of policy texts. (3) Studies tend to focus on single domains, resulting in limited generalizability of findings. Research concentrating on areas like territorial spatial planning and urban planning is gradually increasing. Overall, China’s health impact assessment remains in an exploratory phase, plagued by multiple challenges including inadequate organizational safeguards, insufficient technical capacity reserves, and limited public participation. Pilot projects have seen scant research evaluating actual policy implementation outcomes, failing to meet the practical needs for assessment and summarization during the pilot phase. This hinders efforts to resolve policy implementation challenges encountered during piloting and impedes the comprehensive advancement of future work.

This paper summarizes local government policy implementation issues during the pilot phase through comprehensive policy quantification research, and proposes suggestions and references to promote the important role of health impact assessment in China’s health governance. This will help enhance the government’s public health decision-making and modern governance capabilities, which is of great and far-reaching significance for achieving coordinated development of population health and the economy and society, and fulfilling international commitments under the 2030 Agenda for Sustainable Development.

## 3 Research design

### 3.1 Data sources and samples selection

In 2021, China launched a pilot program to establish a health impact assessment system and required the formulation and improvement of pilot program implementation plans, gradually establishing and improving the health impact assessment system. This study focuses on health impact assessment-related policies issued at the municipal level in China between January 2019 and April 2025. Using the keywords “health impact assessment” and “health impact evaluation,” the study conducted searches in the “Peking University Law Database,” government official websites, and health department official websites, with supplementary searches on Baidu. The inclusion criteria were policies highly relevant to health impact assessment, issued by local governments or their affiliated departments, primarily in the form of “opinions,” “plans,” or “notices,” and currently valid. Exclusion criteria included documents that merely mention health impact assessment without substantive content and documents related to warehouse management regulations, bidding announcements, or solicitation of opinions. A total of 30 policy documents were ultimately included (see Table 1). The final selection of 30 policies strives to reflect practices in health impact assessment across different regions of China. Their representativeness is primarily manifested in the following three aspects: First, broad geographic distribution. These cities collectively cover 15 provinces and autonomous regions, including 17 cities in the eastern region, 7 in the central region, and 6 in the western region, ensuring geographical diversity in the policy sample. Second, inclusion of cities at varying levels of economic development. The selection encompasses both megacities like Guangzhou, Shenzhen, and Hangzhou, as well as a substantial number of small and medium-sized cities (28), which constitute the bulk of China’s urban landscape. This prevents research conclusions from being skewed by economic disparities within the sample. Third, it reflects varying governance capacities. The policy texts examined encompass both first-batch pilot cities (18) and non-first-batch pilot cities (12), as well as provincial capitals (6) and non-provincial capitals (24). This effectively demonstrates how local governments’ understanding and design of HIA policies differ based on their starting points and varying levels of governance capability. The volume of policy texts in this study is moderate compared to related research.

**Table 1:** Municipal-level health impact assessment policies in China.

Code	Policy name	Release agency	Date Issued
P1	Notice of the Office of the People’s Government of Shijiazhuang City on Issuing the Implementation Plan for the Construction of the Health Impact Assessment System in Shijiazhuang City	Office of the People’s Government of Shijiazhuang City	2025.01.17
P2	Notice of the Office of the People’s Government of Jiayuguan City on Issuing the Trial Implementation Plan for the Construction of the Health Impact Assessment System in Jiayuguan City	Office of the People’s Government of Jiayuguan City	2024.12.05
P3	Notice of the Office of the People’s Government of Weihai City on Issuing the Pilot Implementation Plan for the Construction of the Health Impact Assessment System in Weihai City	Office of the People’s Government of Weihai City	2024.12.04
P4	Notice from the Office of the People’s Government of Shenzhen Municipality on the Issuance of the Implementation Opinions on the Establishment of the Health Impact Assessment System in Shenzhen Municipality	Office of the People’s Government of Shenzhen City	2024.05.15
P5	Notice from the People’s Government of Pingxiang City on the Issuance of the Implementation Plan for the Construction of the Health Impact Assessment System in Pingxiang City (Trial)	People’s Government of Pingxiang City	2023.12.14
P6	Notice from the People’s Government of Wuhu City on the Issuance of the Pilot Work Plan for the Construction of the Health Impact Assessment System in Wuhu City (Trial)	People’s Government of Wuhu City	2023.12.06
P7	Notice of the Office of the People’s Government of Jingdezhen City on Issuing the Work Plan for the Construction of the Health Impact Assessment System in Jingdezhen City	Office of the People’s Government of Jingdezhen City	2023.10.31
P8	Notice of the Office of the People’s Government of Nanning City on Issuing the Pilot Work Implementation Plan for the Construction of the Health Impact Assessment System in Nanning City (2023–2025)	People’s Government of Nanning City	2023.10.12
P9	Notice of the Office of the People’s Government of Suizhou City on Issuing the Work Implementation Plan for Health Impact Assessment in Suizhou City (Trial)	Office of the People’s Government of Suizhou City	2023.09.12
P10	Notice of the Office of the People’s Government of Guangzhou City on Issuing the Implementation Plan for the Pilot Project on Health Impact Assessment in Guangzhou City	Office of the People’s Government of Guangzhou City	2023.09.04
P11	Notice of the Office of the People’s Government of Sanming City on Issuing the Implementation Plan for the Pilot Project on the Construction of a Health Impact Assessment System in Sanming City (Trial)	Office of the People’s Government of Sanming City	2023.07.20
P12	Notice of the Office of the People’s Government of Baoji City on Issuing the Implementation Plan for the Construction of a Health Impact Assessment System in Baoji City (Trial)	Office of the People’s Government of Baoji City	2023.05.24
P13	Notice of the Office of the People’s Government of Beihai City on Issuing the Implementation Plan for the Construction of a Health Impact Assessment System in Beihai City (Trial)	Office of the People’s Government of Beih	2023.04.27
P14	Notice of the Office of the People’s Government of Fuyang City on Issuing the Pilot Work Plan for the Construction of a Health Impact Assessment System in Fuyang City (Trial)	Office of the People’s Government of Fuyang City	2023.03.10
P15	Notice of the Office of the People’s Government of Dalian City on Issuing the Work Plan for Health Impact Assessment in Dalian City (Trial)	Dalian Municipal Government Office	2023.03.09
P16	Notice of the People’s Government of Huainan City on Issuing the Implementation Plan for the Pilot Work on the Construction of a Health Impact Assessment System in Huainan City (Trial)	Huainan Municipal Government	2023.01.13
P17	Notice of the Office of the People’s Government of Hefei City on Issuing the Pilot Work Plan for the Construction of a Health Impact Assessment System in Hefei City	Hefei Municipal Government Office	2022.12.30
P18	Notice of the Office of the People’s Government of Lishui City on Issuing the Pilot Work Plan for the Construction of a Health Impact Assessment System in Lishui City	Lishui Municipal Government Office	2022.12.27
P19	Notice of the People’s Government of Chuzhou City on Issuing the Pilot Work Plan for the Construction of a Health Impact Assessment System in Chuzhou City (Trial)	Chuzhou Municipal Government	2022.12.12
P20	Notice of the People’s Government of Rizhao City on Issuing the Pilot Work Implementation Plan for Health Impact Assessment in Rizhao City	Rizhao Municipal Government	2022.12.01
P21	Notice of the Office of the People’s Government of Bozhou City on Issuing the Pilot Work Plan for the Construction of a Health Impact Assessment System in Bozhou City	Bozhou Municipal Government Office	2022.11.29
P22	Notice of the Office of the People’s Government of Shaoxing City on Carrying Out Health Impact Assessment Work	Shaoxing Municipal Government Office	2022.11.07
P23	Notice of the Office of the People’s Government of Changchun City on Issuing the Pilot Work Implementation Plan for the Construction of a Health Impact Assessment System in Changchun City (Trial)	Changchun Municipal Government Office	2022.10.28
P24	Notice of the Office of the People’s Government of Hanzhong City on Issuing the Implementation Plan for the National Pilot Work on the Construction of a Health Impact Assessment System in Hanzhong City (Trial)	Hanzhong Municipal Government Office	2022.09.29
P25	Notice of the Office of the People’s Government of Tongren City on Issuing the Pilot Work Plan for the Construction of a Health Impact Assessment System in Tongren City (Trial)	Tongren Municipal Government Office	2022.07.12
P26	Notice of the Office of the People’s Government of Yichang City on Issuing the Pilot Plan for the Construction of a Health Impact Assessment System in Yichang City (Trial)	Yichang Municipal Government Office	2022.07.08
P27	Notice from the Yichun Municipal People’s Government on the Issuance of the Implementation Plan for the Pilot Work on the Construction of the Health Impact Assessment System in Yichun City (Trial)	Yichun Municipal Government	2022.03.14
P28	Notice from the Zhongshan Municipal People’s Government Office on the Issuance of the Pilot Implementation Plan for Health Impact Assessment in Zhongshan City (Trial)	Zhongshan Municipal Government Office	2022.02.16
P29	Notice from the Hengyang Municipal People’s Government on the Issuance of the Implementation Plan for the Pilot Work on Health Impact Assessment in Hengyang City	Hengyang Municipal Government	2021.12.19
P30	Notice from the Hangzhou Municipal People’s Government Office on the Issuance of the Pilot Implementation Plan for Public Policy Health Impact Assessment in Hangzhou City (Trial)	Hangzhou Municipal Government Office	2019.10.29

### 3.2 Identification of the policy text features

**Figure 1:**
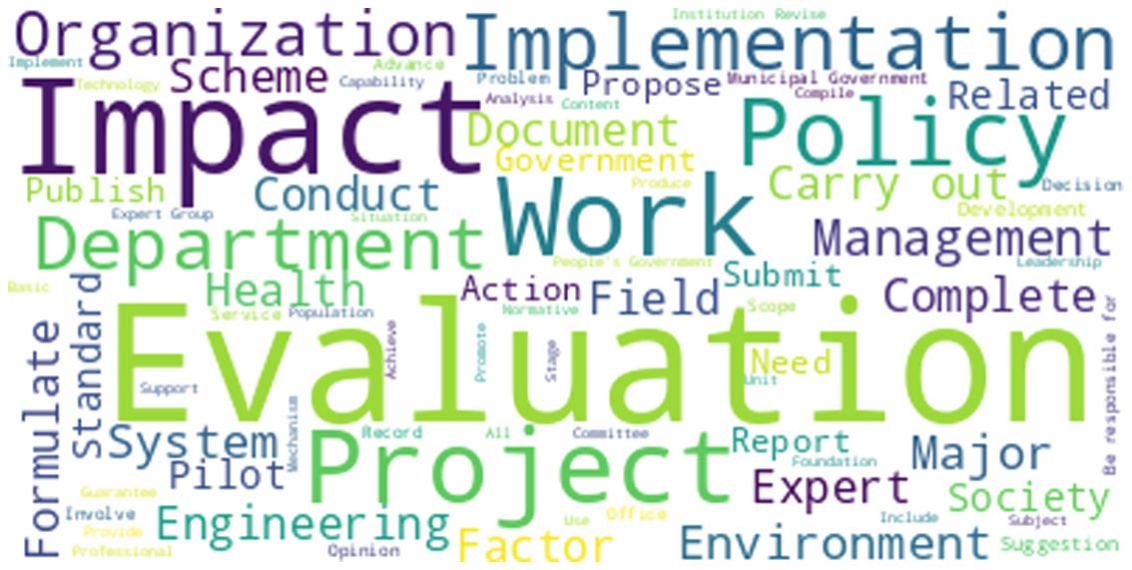
Word cloud of health impact assessment policies.

Using NVivo 11 plus software, we conducted a high-frequency word and word frequency analysis of the policy text content. After removing words with no obvious meaning, such as “at least,” “should,” and “related,” we calculated the top 80 high-frequency words (see Table 2). We then used NVivo 11 plus software to create a word cloud diagram, as shown in Figure 1.

**Table 2:** Hot words and their frequencies in sample policies.

Vocabulary	Frequency	Vocabulary	Frequency	Vocabulary	Frequency	Vocabulary	Frequency
Evaluation	3,183	Promote	327	Issues	179	Leadership	137
Health	3,065	Program	305	Methods	175	Main body	136
Impact	2065	Health	294	Responsibilities	168	Institution	136
Work	1,329	Formulate	277	Services	164	Compilation	133
Project	1,168	Social	276	Promotion	163	Revision	123
Policy	946	Pilot	273	Opinions	162	Content	120
Implementation	824	Standard	255	Decisions	162	Capacity	118
Department	750	Government	252	People’s Government	160	Stage	118
Organization	714	Action	246	Provision	159	Support	118
Management	497	Report	239	Generation	157	Foundation	116
Public	462	Release	228	Committee	156	Unit	115
System	418	Development	222	Technology	155	Promotion	113
Environment	399	Establish	219	Professional	154	Analysis	111
Construction	395	Recommendation	218	Mechanism	151	Situation	109
Major	395	Strengthen	200	Use	150	Implementation	108
Field	357	Municipal Government	194	Regulations	149	Issuance	106
Document	349	Office	190	Population	145	Trial implementation	105
Expert	348	Involve	187	Normative	144	Process	104
Engineering	341	Record	185	Guarantee	142	Administration	102
Factor	327	Scope	181	Expert Group	141	Revision	98

### 3.3 Construction of the PMC index model

The PMC index model is a policy evaluation tool proposed by Estrada et al. (34) based on the OmniaMobilis hypothesis, which argues that policy research models should strive to broaden their scope and ensure the comprehensiveness of variable indicators (12) in order to scientifically evaluate the consistency between policies and intuitively reflect their advantages and disadvantages.

#### 3.3.1 Variable classification and parameter identification

To establish an indicator system, this study simultaneously employed text mining and literature review methods, drawing extensively on existing high-frequency terms and relevant research. We established nine primary variables: Policy Nature (X1), Policy Timeliness (X2), Policy Subject (X3), Policy Object (X4), Policy Content (X5), Policy Domain (X6), Safeguard Measures (X7), Policy Tools (X8), and Policy Evaluation (X9). By extracting key variables from relevant literature and policies and defining sub-variables, we identified 39 sub-variables. Two individuals with experience in health impact assessment worked on constructing the indicators, and any inconsistencies were resolved by health impact assessment experts. Ultimately, nine first-level variables and 39 second-level variables were finalized, as shown in Table 3.

**Table 3:** Quantity indicators and parameter settings.

Primary variable	Secondary variable	Evaluation scale	Reference
X1 policy nature	X1-1 Prediction	Is it predictive? Based on data analysis and trend research, anticipate future issues and formulate measures in advance.	Estrada (12)
	X1-2 Recommendation	Is it advisory? Provide reference opinions based on feedback from experts or stakeholders.	
	X1-3 Supervision	Is it regulatory? Set rules and standards to ensure compliance.	
	X1-4 Description	Explain the policy background, objectives, and measures in detail to help the public understand.	
	X1-5 Guidance	Clarify the policy direction and objectives to guide subsequent implementation.	
X2 policy validity period	X2-1 Long-term	Does it involve a plan spanning more than five years?	Yu Xue et al. (13)
	X2-2 Medium-term	Does it involve a plan spanning three to five years?	
	X2-3 Short-term	Does it involve a plan spanning one to two years?	
X3 policy subject	X3-1 Government	Is the issuing authority the government?	Wu Weihong et al.(40)
	X3-2 Government Office	Is the issuing authority the government office (bureau)?	
X4 policy object	X4-1 Government	Does the policy subject involve the government?	Xue Huiyuan, Zhang Yonggao (41)
	X4-2 Public	Does the policy subject involve the public?	
	X4-3 Experts and technical personnel	Does the policy subject involve experts or technical personnel?	
	X4-4 Third-party institutions	Does the policy subject involve third-party institutions?	
X5 policy content	X5-1 Determine the scope of assessment	Does it include content such as health impact assessments for important public policies, major construction projects, and related major plans?	Knowledge network graph based on text mining
	X5-2 Clarify the assessment content	Does it include assessments based on major health impact factors such as material environmental factors, social environmental factors, biological and psychological factors, lifestyle factors, and healthcare service factors, as well as physical health, mental health, social adaptation, and moral health?	
	X5-3 Improve the assessment process	Does it include assessment methods, implementation pathways, and technical routes?	
	X5-4 Strengthen organizational leadership	Does it include provisions clarifying the responsible entities, implementing entities, and executing entities for health impact assessments?	
	X5-5 Improve operational mechanisms	Does it include provisions on task division, work networks, and coordination mechanisms?	
	X5-6 Enhance support measures	Does it include provisions related to talent development, financial investment, monitoring and evaluation, supervision and assessment, technical support, legal safeguards, training and publicity, establishing exemplary cases, and collaborative efforts?	
X6 policy area	X6-1 Administrative normative documents	Does it include administrative normative documents?	Based on text mining
	X6-2 Engineering projects	Does it include engineering projects?	
	X6-3 Spatial planning	Does it include spatial (urban) planning?	
X7 guarantee measures	X7-1 Talent development	Does it include measures for talent development?	Gu Yichun et al. (42) and Jia Tingting et al. (43)
	X7-2 Fiscal investment	Does it include budgetary provisions to ensure funding for related expenses?	
	X7-3 Monitoring and evaluation	Does it include monitoring and evaluation of the implementation process and post-implementation outcomes of the assessment project?	
	X7-4 Supervision and assessment	Does it establish a target-based performance evaluation indicator system or supervision mechanism?	
	X7-5 Technical support	Does it include provisions related to technical support and the application of new technologies?	
	X7-6 Legal safeguards	Does it include legal safeguards?	
	X7-7 Training and publicity	Are there requirements and measures to enhance the government’s and the public’s awareness of and participation in health impact assessment work?	
	X7-8 Setting examples	Are pilot projects and exemplary demonstrations encouraged?	
	X7-9 Division of labor and collaboration	Is there a clear mechanism for the division of responsibilities and interdepartmental collaboration?	
X8 policy tools	X8-1 Supply-oriented tools	As a resource provider, the government meets the needs of target groups by directly increasing resource supply, improving infrastructure, or providing public services, thereby promoting the achievement of health impact assessment policy objectives. This primarily includes “blood transfusion” methods such as talent cultivation, financial investment, and technical support.	Zhang Xiaojie et al. (44)
	X8-2 Demand-oriented tools	The government addresses the demand side of health impact assessment actions by stimulating the demand or altering the behavior of various entities such as the government and the public, thereby driving the development of related products or services. Through “demand-driven” approaches, it indirectly achieves policy objectives by optimizing supply. This primarily includes demonstration pilot projects, prioritizing policies or projects with no health impacts, and regularly monitoring public opinion.	
	X8-3 Environment-oriented tools	By altering the policy environment (such as regulations, standards, and information provision), the government indirectly guides individual or organizational behavior to achieve policy objectives, creating an “invisible hand” to provide a favorable policy environment for health impact assessment initiatives. This primarily includes goal planning, policy regulation, legal oversight, and standard evaluation.	
X9 policy evaluation	X9-1 Sufficient basis	Is there sufficient basis for formulation?	Xu Pingping et al. (45) and Xiao XingYi et al. (46)
	X9-2 Clear objectives	Are there clear policy objectives?	
	X9-3 Scientific plans	Is there a scientific policy plan?	
	X9-4 Clear responsibilities and authorities	Is there a clear division of responsibilities?	

#### 3.3.2 Reliability assessment of coding

To assess the reliability of variable coding in the PMC indicator model, we conducted an inter-rater reliability analysis. Two researchers independently coded 10 policy texts (representing 33% of the total sample: P1, P4, P5, P10, P15, P16, P18, P20, P28, P29) using Excel’s random function for selection. Intraclass correlation coefficients (ICCs) were calculated using a two-way random-effects model via the SPSS PRO online statistical analysis platform (Standard Edition), with absolute agreement metrics reported. Analysis results showed an ICC of 0.78 (95% CI: 0.705–0.854) for the single indicator, with a *p* < 0.001. This value exceeded the 0.75 threshold, indicating “good” inter-rater reliability. This statistical validation confirms the high stability and reproducibility of the variable coding process in this study.

#### 3.3.3 Building a multi-input–output table

A multi-input–output table is the basis for calculating the PMC index. Essentially, it is a data analysis framework that can store large amounts of data and perform quantitative analysis of a single variable from multiple angles. Constructing a multi-input–output table facilitates a more comprehensive and intuitive evaluation of the policy under consideration. By integrating the variable indicator system established in the preceding section with the inherent characteristics of the policy, this study constructs a multi-input–output table comprising 9 primary variables and 39 secondary variables for the policy (see Table 4). The study employs a binary method to assign equal weights to all sub-variables. If a specific strategy aligns with a given sub-variable, its parameter is set to 1; otherwise, it is set to 0.

**Table 4:** Multi-input–output table.

Primary variable	X1	X2	X3	X4	X5	X6	X7	X8	X9
Secondary variable	X1-1	X2-1	X3-1	X4-1	X5-1	X6-1	X7-1	X8-1	X9-1
	X1-2	X2-2	X3-2	X4-2	X5-2	X6-2	X7-2	X8-2	X9-2
	X1-3	X2-3		X4-3	X5-3	X6-3	X7-3	X8-3	X9-3
	X1-4			X4-4	X5-4		X7-4		X9-4
	X1-5				X5-5		X7-5		
					X5-6		X7-6		
							X7-7		
							X7-8		
							X7-9		

#### 3.3.4 Measurement of the PMC-index

Firstly, according to Ruiz Estrada’s method, the binary method is used to assign values to secondary variables according to Equations 1, 2. Then, the values of first-level variables for each policy are calculated according to Equation 3, and the PMC index for each policy is calculated according to Equation 4.


(1) X∼N≤[0,1]

(2) X={XR:[0,1]}

(3) $$X_{i}=\sum_{j=1}^{n}\frac{X_{\mathrm{ij}}}{n(X_{\mathrm{ij}})}$$

(4) $$\begin{array}{l} \;\;\mathrm{PMC}=[X_{1}(\sum_{t=1}^{5}\frac{X_{1t}}{5})+X_{2}(\sum_{j=1}^{3}\frac{X_{2j}}{3})+X_{3}(\sum_{k=1}^{2}\frac{X_{3k}}{2})+X_{4}(\sum_{l=1}^{4}\frac{X_{4l}}{4})\\ +X_{5}(\sum_{m=1}^{6}\frac{X_{5m}}{6})+X_{6}(\sum_{n=1}^{3}\frac{X_{6n}}{3})+X_{7}(\sum_{o=1}^{9}\frac{X_{7o}}{9})\\ +X_{8}(\sum_{p=1}^{3}\frac{X_{8p}}{3})+X_{9}(\sum_{q=1}^{4}\frac{X_{9q}}{4})] \end{array}$$

In Equations 1, 2: t is a primary variable (taking integer values between 1 and 9), i is a secondary variable, and n denotes the number of secondary variables corresponding to a given primary variable (35).

Based on the evaluation criteria established by Estrada (11) and other scholars, a PMC score between (7, 10) indicates that the policy is comprehensive and reasonable; PMC scores between [5, 6.99] indicate an acceptable policy; PMC scores between [3, 4.99] indicate an unbalanced policy; PMC scores between [0, 2.99] indicate an ineffective policy. These are categorized into four policy grades.

#### 3.3.5 Policy deficiency index

To highlight the shortcomings of policies and explore optimization pathways for health impact assessment policies, this study introduces the Policy Deficiency Index. The Policy Deficiency Index = 10 – PMC Index. Based on the calculated PMC Index, this study constructs a 3 × 3 matrix and plots the PMC surface diagrams for each policy (35).

A concavity index between (1, 2) is evaluated as low concavity, between (2, 4) as moderate concavity, between (4, 6) as acceptable concavity, and between (6, 10) as unacceptable concavity.

#### 3.3.6 Construction of the PMC surface

Finally, draw a PMC surface graph according to Equation 5 to more intuitively display the advantages and disadvantages of the holistic and individual policies. The PMC surface diagram visually presents the evaluation results of the PMC index model. The smoothness and height of the surface diagram reflect the quality of the policy; the smoother the surface and the higher the height, the better the policy quality (35).


(5) $$\mathrm{PMC}=\begin{array}{ccc} X_{1} & X_{4} & X_{7}\\ X_{2} & X_{5} & X_{8}\\ X_{3} & X_{6} & X_{9} \end{array}$$

## 4 Results and analysis

### 4.1 Holistic evaluation of policies

The results of the calculation of the PMC index for health impact assessment policies are shown in Supplementary Table S1 and Table 5. Among the 30 health impact assessment policies, 11 were complete and reasonable, and 19 were acceptable. The quality of the policies was at an acceptable level or above. The average PMC index score for the policies was 6.89, with all scores at or above 6.36. The average concavity index was 3.11, classified as moderate concavity, above the acceptable level. Overall, it indicates that existing HIA policies are generally effective, providing a fundamental framework and impetus for pilot initiatives while ensuring their orderly implementation. However, the “central dip” also reveals imbalances in policy coverage, suggesting notable shortcomings in specific dimensions such as policy effectiveness, safeguards, or coverage areas, which constrain the full realization of policy efficacy.

**Table 5:** PMC-index and level of policies.

Code	X1	X2	X3	X4	X5	X6	X7	X8	X9	PMC index	Rank	deficiency Index
P1	1.00	0.33	0.50	1.00	1.00	1.00	0.78	0.67	1.00	7.28	4	2.72
P2	1.00	0.33	0.50	0.25	1.00	1.00	0.67	0.67	1.00	6.42	29	3.58
P3	1.00	0.33	0.50	1.00	1.00	1.00	0.56	1.00	1.00	7.39	3	2.61
P4	1.00	0.33	0.50	0.50	1.00	1.00	0.78	1.00	1.00	7.11	8	2.89
P5	1.00	0.33	0.50	1.00	1.00	1.00	0.78	0.67	1.00	7.28	4	2.72
P6	1.00	0.33	0.50	0.75	1.00	1.00	0.44	0.67	1.00	6.69	23	3.31
P7	1.00	0.33	0.50	0.75	1.00	1.00	0.56	0.67	1.00	6.81	19	3.19
P8	1.00	0.33	0.50	0.50	1.00	1.00	0.56	0.67	1.00	6.56	28	3.44
P9	1.00	0.33	0.50	0.75	1.00	0.67	0.44	0.67	1.00	6.36	30	3.64
P10	1.00	0.33	0.50	0.75	1.00	0.67	0.44	0.67	1.00	6.36	30	3.64
P11	1.00	0.33	0.50	0.75	1.00	0.67	0.89	0.67	1.00	6.81	18	3.19
P12	1.00	0.33	0.50	0.75	1.00	1.00	0.56	0.67	1.00	6.81	19	3.19
P13	1.00	0.33	0.50	0.75	1.00	1.00	0.78	0.67	1.00	7.03	10	2.97
P14	1.00	0.33	0.50	0.75	1.00	0.67	0.67	0.67	1.00	6.58	26	3.42
P15	1.00	0.33	0.50	1.00	1.00	0.67	0.56	0.67	1.00	6.72	21	3.28
P16	1.00	0.33	0.50	1.00	1.00	1.00	0.56	0.67	1.00	7.06	9	2.94
P17	1.00	0.33	0.50	1.00	1.00	1.00	0.44	0.67	1.00	6.94	12	3.06
P18	1.00	0.33	0.50	0.75	1.00	0.67	0.78	0.67	1.00	6.69	23	3.31
P19	1.00	0.33	0.50	0.75	1.00	0.67	0.67	0.67	1.00	6.58	26	3.42
P20	1.00	0.33	0.50	1.00	1.00	0.67	0.78	0.67	1.00	6.94	12	3.06
P21	1.00	0.33	0.50	1.00	1.00	0.67	0.67	0.67	1.00	6.83	16	3.17
P22	1.00	0.33	0.50	0.75	1.00	0.67	0.78	1.00	1.00	7.03	10	2.97
P23	1.00	0.33	0.50	1.00	1.00	0.67	0.89	1.00	1.00	7.39	1	2.61
P24	1.00	0.33	0.50	1.00	1.00	1.00	0.67	0.67	1.00	7.17	6	2.83
P25	1.00	0.33	0.50	1.00	1.00	0.67	0.78	0.67	1.00	6.94	12	3.06
P26	1.00	0.33	0.50	1.00	1.00	0.67	0.56	0.67	1.00	6.72	21	3.28
P27	1.00	0.33	0.50	1.00	1.00	0.67	0.89	1.00	1.00	7.39	1	2.61
P28	1.00	0.33	0.50	0.75	1.00	1.00	0.44	0.67	1.00	6.69	23	3.31
P29	1.00	0.33	0.50	1.00	1.00	1.00	0.67	0.67	1.00	7.17	6	2.83
P30	1.00	0.33	0.50	1.00	1.00	0.67	0.67	0.67	1.00	6.83	16	3.17
Average	1.00	0.33	0.50	0.84	1.00	0.83	0.66	0.72	1.00	6.89		3.11

From the perspective of the mean scores of first-level variables, the mean score for policy nature (X1) was 1, encompassing predictive, advisory, regulatory, descriptive, and directive content; the mean score for policy timeliness (X2) was 0.33, with most policies being medium- to short-term plans and guidelines (18 policies) rather than long-term plans (12 policies); The average score for policy subject (X3) is 0.5, with a higher number of policies issued by government offices (23 items) than those issued in the name of the government (7 items); The average score for policy object (X4) is 0.84, with many policies mentioning government officials, experts, and third-party institutions, but relatively fewer mentioning the public (18 items); The average score for policy content (X5) is 1, with policies covering the determination of assessment scope, clarification of assessment content, improvement of assessment processes, strengthening of organizational leadership, improvement of operational mechanisms, and enhancement of support measures; The average score for policy areas (X6) is 0.83, with most policies involving administrative normative documents and engineering projects, but relatively few spatial planning policies (15 items); The average score for support measures (X7) is 0.66. Most policies involve talent cultivation, monitoring and evaluation, fiscal investment, technical support, training and publicity, and division of labor and collaboration, but there are few measures related to supervision and evaluation (15 items), legal safeguards (0 items), and setting examples (5 items); The average score for policy tools (X8) was 0.72, with the three types of policy tools primarily focused on supply-oriented and environmental tools, while demand-oriented tools were relatively scarce (5 items); The average score for policy evaluation (X9) was 1, with all policies meeting the evaluation criteria of adequate basis, clear objectives, scientific plans, and clear responsibilities.

Overall, among all average scores, only the policy timeliness score was relatively low (0.33). First, this reflects that local governments, lacking mature experience, tend to formulate short-term plans or guidelines with a duration of 1–3 years. This strategy grants policies greater flexibility, facilitating rapid adjustments and corrections based on practical outcomes. Second, this short-term orientation also reveals potential resource constraints and challenges in allocating responsibilities for HIA work. Medium-to-short-term planning to some extent reflects that current HIA resource allocation (such as funding and personnel) remains informal. The authority and coordination capabilities of the lead department (typically the health department) may be limited, making it difficult to establish and fulfill long-term commitments spanning 5 years or more.

### 4.2 Specific evaluation of policies

**Figure 2:**
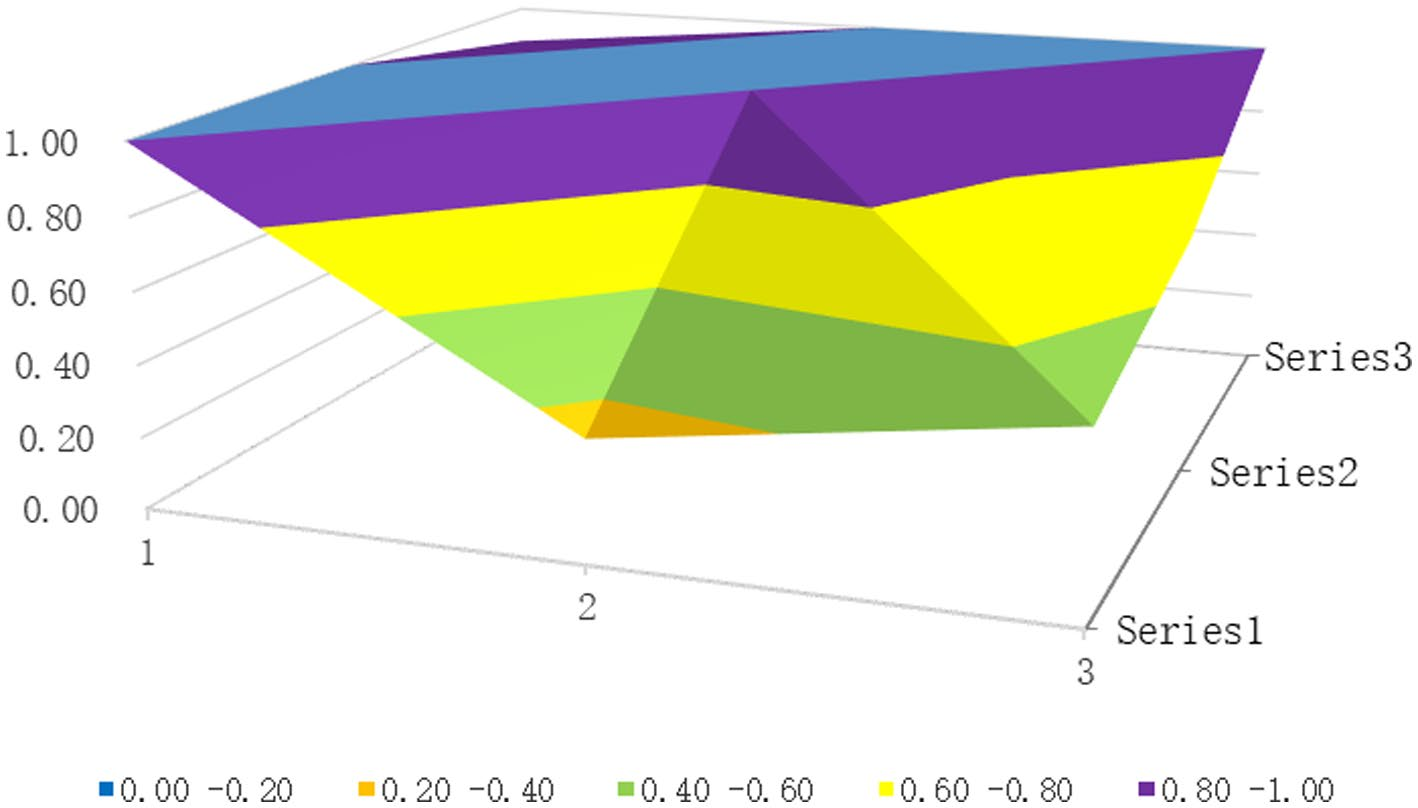
PMC surface graph of P23.

**Figure 3:**
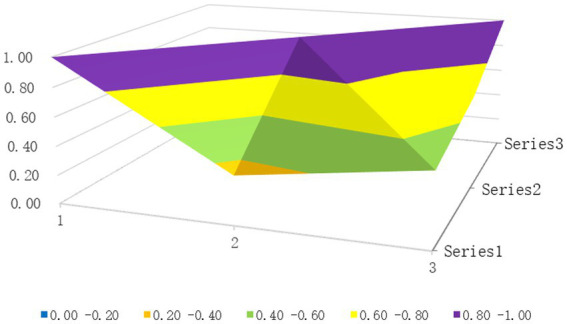
PMC surface graph of P9.

The surface diagrams of the two policies with the highest and lowest PMC index scores, P23 and P9, respectively, are selected (see Figures 2, 3). Different colors on the PMC surface diagram represent the scores of different variable indicators, and the advantages and disadvantages of the policies can be judged by the degree of concavity and convexity of the surface.

The PMC index of the P23 policy is 7.39, and the policy rating is complete and reasonable. The PMC surface diagram shows minimal fluctuations, with high scores across all dimensions, as shown in Figure 2. Among the nine primary indicators, five scored 1 point each. Only the policy timeliness and policy domain indicators exhibit significant concavity: policy timeliness is short-term, and the policy domain does not include spatial planning. This policy involves predictive, advisory, regulatory, descriptive, and directive content; it was issued by the government office. The policy subject, policy content, policy tools, and policy evaluation are comprehensive, covering all secondary indicators. The safeguards include talent cultivation, fiscal investment, monitoring and evaluation, supervision and assessment, technical support, training and publicity, setting examples, and division of labor and collaboration. This indicates that its various functions are complete, taking into account the connotations of all areas of health impact assessment, and can effectively promote the implementation of health impact assessment.

The PMC index for Policy P9 is 6.36, with a policy rating of acceptable. The PMC surface plot shows minimal fluctuations, as shown in Figure 3. This policy was issued by the government, with a short-term policy timeline. The policy domain does not include spatial planning, and the policy subject does not involve third-party evaluation institutions; the policy nature, policy content, and policy evaluation are comprehensive, covering all secondary indicators; the safeguards include only talent development, technical support, training and publicity, and collaboration; and the policy tools do not include demand-driven tools. The P9 policy indicates that a high-quality HIA policy should be a comprehensive institutional arrangement characterized by long-term effectiveness, coverage of key areas, mobilization of diverse stakeholders, and the use of a hybrid toolkit.

### 4.3 Policy evaluation of health impact assessment in pilot cities and non-pilot cities

The cities covered by the policy text of this study were divided into pilot cities and non-pilot cities, and the average PMC index values were compared. The average values for the first batch of pilot cities and non-first batch pilot cities were 6.89 and 6.82, respectively. The first batch of pilot cities scored higher than the non-first-batch pilot cities, indicating that the balance of policy levels between cities needs to be improved. See Table 6 for details.

**Table 6:** Comparison of PMC index between pilot cities and non-pilot cities.

Classification	Policy	PMC Index	Average
Pilot cities	P1	7.28	6.89
	P8	6.56	
	P15	6.72	
	P20	6.94	
	P23	7.39	
	P24	7.17	
	P25	6.94	
	P26	6.72	
	P27	7.39	
	P28	6.69	
	P29	7.17	
	P30	6.83	
Non-pilot cities	P2	6.42	6.82
	P3	7.39	
	P4	7.11	
	P5	7.28	
	P6	6.69	
	P7	6.81	
	P9	6.36	
	P10	6.36	
	P11	6.81	
	P12	6.81	
	P13	7.03	
	P14	6.58	
	P16	7.06	
	P17	6.94	
	P18	6.69	
	P19	6.58	
	P21	6.83	
	P22	7.03	

## 5 Discussion and conclusion

### 5.1 Conclusion

This study offers a comprehensive evaluation of municipal-level Health Impact Assessment (HIA) policies in China by integrating text mining and a structured PMC (Policy Modeling Consistency) index framework. Through the analysis of 30 representative policy documents, our findings reveal that the overall design of HIA policies is relatively robust, with most policies reaching acceptable or higher levels of consistency. High scores were observed in areas such as policy nature, content, and evaluation mechanisms, indicating a solid foundation for health policy integration at the municipal level. These policies have played an active role in supporting the goal of incorporating health into all policies and promoting population well-being.

However, the results also highlight significant structural and operational shortcomings that warrant attention. Low-scoring dimensions such as timeliness, issuing bodies, supportive measures, and policy tools point to a lack of institutional continuity, weak enforcement mechanisms, and insufficient intersectoral collaboration. Furthermore, the average concavity index suggests that many policies fall short of optimal consistency, reflecting the uneven development of HIA frameworks across different regions. This indicates that since the pilot program began in 2021, the health impact assessment policies introduced have generally facilitated the orderly advancement of the pilot work. However, targeted refinements are still needed to address identified shortcomings, thereby enhancing the overall effectiveness and balance of the policies.

To address these limitations, several key directions for policy improvement are recommended. First, it is essential to strengthen top-level policy design by embedding HIA requirements into national legal and regulatory systems, ensuring long-term stability and enforceability. Second, the establishment of a comprehensive supervision and evaluation mechanism will be crucial in enhancing accountability and promoting implementation fidelity. Third, diversifying the policy toolset - including financial incentives, administrative guidance, and cross-sectoral integration – can enhance the responsiveness and adaptability of HIA frameworks. In addition, fostering interregional exchange and learning can help bridge capacity disparities and promote the diffusion of best practices, thereby contributing to more balanced development of HIA systems nationwide. Ultimately, by adopting a more systemic, evidence-informed, and future-oriented approach to HIA policymaking, Chinese municipalities can better align with global standards and more effectively contribute to the long-term objectives of health equity, policy efficiency, and sustainable development.

### 5.2 Implications

#### 5.2.1 A legal framework for health impact assessment urgently needs to be established

This recommendation stems from low policy timeliness scores (X2, mean = 0.33) and the complete absence of legal safeguards across all policies (sub-variable under X7). At the municipal level, short-term, non-mandatory regulatory documents are prevalent, reflecting insufficient institutional continuity and enforceability. Our analysis confirms that none of the policies incorporate legal protection mechanisms. Only a few regions have made scattered references or provisions related to health impact assessments in other local regulations or rules. This reflects a significant shortfall in the legal support for China’s health impact assessment (HIA) policies, and there is an urgent need to strengthen the policy framework. Although the Basic Medical and Health Promotion Law of the People’s Republic of China, enacted in 2019, provides a certain legal foundation for health impact assessment work as a legislative document, it is insufficient to comprehensively promote the implementation of the health-first development strategy by all regions and departments, integrate health protection deeply into the socioeconomic policy objectives system, or effectively prevent major health and safety risks, thereby constraining the sustained improvement of public health levels. At the national level, there is a need to strengthen top-level design, establish a dedicated task force for the formulation of health impact assessment regulations, and accelerate the legislative process. However, the feasible path to advancing HIA legislation requires careful design. Relying solely on the direct enactment of high-level laws at the national level may encounter resistance due to insufficient practical experience. Therefore, the legal framework for HIA must be built upon a pilot model. This approach has recently gained policy validation and practical implementation. In early 2025, the National Health and Family Planning Commission summarized and reported on HIA pilot initiatives, directing all regions to comprehensively advance institutional development. It explicitly designated cities with strong foundational work—including Tianjin, Chongqing, Hangzhou, Ma’anshan, Yichang, and Panzhihua—as “legislative liaison points.” This signifies central recognition of the necessity for local legislative precedents, initiating organized and selective encouragement and support for mature cities to pioneer legislation. The existing laws, such as the Basic Medical and Health Care and Health Promotion Law of the People’s Republic of China, can serve as a basis, drawing on international best practices and successful cases from other regions within China. The content should clearly define the scope, process, standards, and responsible entities of health impact assessment, providing clear and unified legal guidance for such work.

This study also reveals a predominance of short- and medium-term policies. Future optimization of HIA policies must focus on transitioning from “short-term pilot programs” to “long-term institutionalization.” However, this shift should not rely solely on increasing the number of long-term policies. It is equally crucial to establish strategic long-term plans at the national or provincial level, providing local governments with clear top-level design and stable policy expectations. Policy texts should strengthen provisions for resource allocation to ensure long-term objectives are supported by adequate financial, human, and institutional resources. The legal status of HIA within major administrative decision-making processes must be clarified, elevating its policy effectiveness level. This will encourage local governments to treat HIA as a foundational undertaking requiring sustained investment rather than a temporary task.

#### 5.2.2 Constraint and supervision mechanisms urgently need improvement

This is necessitated by the identified weakness in safeguard measures (X7, mean = 0.66), where only half of the policies contained supervision and evaluation content. This structural gap risks rendering well-designed policies ineffective during implementation. Currently, health impact assessment policies are still in the pilot stage and involve multiple interest groups, so effective supervision and evaluation methods and approaches are still being explored. Supervision and evaluation, as a critical link in ensuring the implementation of policies, play an irreplaceable role in ensuring the quality and effectiveness of health impact assessment work. On one hand, it is necessary to establish and improve the supervision mechanism for health impact assessment, incorporate health impact assessment work into the performance evaluation system of relevant departments and personnel, clarify evaluation indicators and weights, and conduct quantitative evaluations of the completion status and quality of assessment work to form an effective incentive and constraint mechanism, fully mobilizing the initiative and proactivity of relevant departments and personnel in conducting health impact assessment work. For example, some cities, such as Yichun and Jingdezhen, have proposed gradually exploring the formulation and improvement of various supervision, performance evaluation methods, and incentive mechanisms for health impact assessment work and have incorporated the conduct of health impact assessment work into the local healthy city evaluation system, effectively leveraging the supervisory and guiding role of evaluations (36, 37). On the other hand, efforts should be made to enhance public awareness and attention to health impact assessments through strengthened public outreach, fostering a favorable societal environment where all sectors collectively focus on the health impacts of public policies, thereby laying a solid public foundation for the smooth implementation of health impact assessment work. For example, cities like Yichun have actively explored the establishment of effective public participation mechanisms for health impact assessments, encouraging the public to monitor potential negative health impacts resulting from policy implementation and fully leveraging the supervisory role of the public in health impact assessment work (36).

#### 5.2.3 Policy tool structure urgently needs optimization

This imperative stems from the severe deficiency in demand-oriented tools (a secondary variable under X8), which were present in only 5 out of 30 policies. This over-reliance on supply-oriented and environment-oriented tools fails to mobilize non-governmental actors. Demand-type tools refer to government interventions targeting the demand side of the Healthy China Initiative, aiming to provide a driving force for achieving policy objectives and reduce resistance during policy implementation (38). Since health impact assessment work in China is primarily government-led, public participation is relatively low, resulting in a limited range of policy tools being used. Policy tool theory suggests that the reasonable and balanced use of environmental, supply-side, and demand-side policy tools is key to maximizing policy effectiveness and ensuring effective government governance (39). The use of demand-side policy tools should be strengthened in a timely manner based on the progress and effectiveness of policy implementation. First, the promotion of typical cases should be intensified. For outstanding practice cases and successful experiences identified in health impact assessment work, they should be promptly reported to the Health China Action Promotion Office and the National Health Education Office to carry out the promotion and application of assessment outcomes. Units that actively adopt these outstanding outcomes should be provided with policy support and resources to incentivize more cities to proactively participate in health impact assessment work. For example, Shaoxing City has proposed to establish around 10 municipal-level outstanding cases by the end of 2023; around 20 municipal-level outstanding cases by the end of 2024, with the aim of having outstanding cases selected as provincial-level models; and a batch of provincial-level or higher health impact assessment models by the end of 2025. Through this approach, the exemplary and leading role of outstanding cases should be fully leveraged to promote the expansion of health impact assessment work on a larger scale. Second, it is necessary to effectively enhance awareness of “integrating health into all policies” and inject strong momentum into health impact assessment work from the demand side. Through publicity and advocacy, pre-service and in-service training for officials, and other means, the strategy of “integrating health into all policies” should be applied to addressing health issues, fully recognizing the importance of fulfilling responsibilities for the health of the people, and actively and proactively conducting health impact assessments of public policies and major livelihood projects (36).

#### 5.2.4 Interregional exchange and cooperation urgently need to be strengthened

By comparing the average PMC index values of the first batch of pilot cities (mean PMC = 6.89) and non-pilot cities(mean PMC = 6.82), significant differences were found in policy areas, safeguards, and policy tools, revealing an uneven development where the first batch of pilot cities outperformed non-pilot cities. For example, Zhejiang, Shijiazhuang, and Yichun have taken the lead in health impact assessment work, proposing a number of practical and feasible safeguards and mechanisms; in contrast, policies in non-first-batch pilot cities are relatively conservative, largely following national guiding documents in design, lacking innovation and specificity. To narrow regional development gaps and promote balanced development in health impact assessment work, it is recommended to establish a regional exchange and learning platform for health impact assessment, organizing diverse talent exchange activities such as regular seminars, forums, and training programs. These initiatives should provide regions with ample opportunities to exchange experiences, share outcomes, and discuss challenges, fostering experience sharing, complementary strengths, and collaborative development in health impact assessment work across regions, thereby enhancing the professional standards of such work. Additionally, it is recommended to explore the establishment of a regional mutual assistance mechanism, encouraging developed regions or those with more advanced assessment capabilities to provide guidance and support to relatively underdeveloped regions, sharing assessment experiences, technical methods, and resources to help underdeveloped regions enhance their health impact assessment capabilities. Furthermore, it is advisable to encourage regions to collaborate on joint projects, conducting health impact assessment research and practice together to achieve complementary advantages and resource sharing, thereby driving the overall improvement of China’s health impact assessment work.

### 5.3 Limitations and further works

Despite providing a comprehensive evaluation of municipal-level Health Impact Assessment (HIA) policies in China through the PMC index model, this study has several limitations that warrant further exploration. First, the policy sample is limited to 30 municipal-level documents issued since 2019, which may not fully reflect the diversity of HIA practices across different administrative levels or regions, particularly in economically less developed areas. Future studies should consider expanding the dataset to include provincial-level regulations, local pilot policies, and interdepartmental guidance documents to capture a more representative spectrum of HIA policy implementation nationwide.

Second, although the indicator system was developed based on high-frequency terms from text mining, content analysis, and expert consultation, the classification and weighting of both primary and secondary variables inevitably involve a degree of subjectivity. To enhance the objectivity and theoretical rigor of future evaluations, future research may employ methods such as the Delphi technique and coder reliability testing to enhance robustness.

Third, while the PMC index model enables systematic comparison of policy structure and consistency, its analytical focus remains confined to policy texts. It does not account for the real-world effectiveness of policy implementation or the extent to which intended health outcomes have been achieved. This methodological limitation suggests a need for future research to integrate complementary approaches—such as field surveys, case studies, or policy outcome evaluations—to bridge the gap between textual content quality and actual policy performance.

Lastly, this study did not explore the longitudinal evolution of HIA policy content or its responsiveness to public health emergencies, environmental transitions, or social changes. Future work could incorporate a dynamic temporal dimension to examine how HIA policies adapt over time, thereby offering insights into policy resilience and strategic alignment with national health goals.

## Data Availability

The original contributions presented in the study are included in the article/Supplementary material, further inquiries can be directed to the corresponding author.
